# Efficacy of contact lenses for myopia control: Insights from a randomised, contralateral study design

**DOI:** 10.1111/opo.13042

**Published:** 2022-08-25

**Authors:** Rebecca Weng, Weizhong Lan, Ravi Bakaraju, Fabian Conrad, Thomas Naduvilath, Zhi‐kuan Yang, Padmaja Sankaridurg

**Affiliations:** ^1^ Brien Holden Vision Institute Sydney New South Wales Australia; ^2^ Aier Institute of Optometry and Vision Science Changsha China; ^3^ Aier School of Ophthalmology Central South University Changsha China; ^4^ School of Optometry and Vision Science University of New South Wales Sydney New South Wales Australia

**Keywords:** contact lenses, contralateral, cross‐over, dual focus, extended depth of focus, myopia progression

## Abstract

**Purpose:**

To determine the efficacy of two myopia control contact lenses (CL) compared with a single‐vision (SV) CL.

**Methods:**

Ninety‐five Chinese children with myopia, aged 7–13 years in a 1‐year prospective, randomised, contralateral, cross‐over clinical trial with 3 groups; bilateral SVCL (Group I); randomised, contralateral wear of an extended depth of focus (EDOF) CL and SVCL (Group II) and MiSight® CL and SVCL (Group III). In Groups II and III, CL were crossed over at the 6‐month point (Stage 1) and worn for a further 6 months (Stage 2). Group I wore SVCL during both stages. At baseline and the end of each stage, cycloplegic spherical equivalent refractive error (SE) and axial length (AL) were measured. Six‐monthly ΔSE/ΔAL across groups was analysed using a linear mixed model (CL type, stage, eye and eye* stage included as factors). Intra‐group paired differences between eyes were determined.

**Results:**

In Group I, mean (SD) ΔSE/ΔAL with SVCL was −0.41 (0.28) D/0.13 (0.09) mm and −0.25 (0.27) D/0.16 (0.09) mm for stages 1 and 2, with a mean paired difference between eyes of 0.01 D/0.01 mm and 0.05 D/−0.01 mm, respectively. ΔSE/ΔAL with SVCL was similar across Groups I to III (Stage 1: *p* = 0.89/0.44, Stage 2: *p* = 0.70/ 0.64). In Groups II and III, ΔSE/ΔAL was lower with the EDOF and MiSight® CL than the contralateral SVCL in 68% to 94% of participants, and adjusted 6‐month ΔSE/ΔAL with EDOF was similar to MiSight® (*p* = 0.49/0.56 for ΔSE/ΔAL, respectively). Discontinuations across the three groups were high, but not different between the groups (33.3%, 48.4% and 50% for Groups I to III, respectively [*p* = 0.19]) and most discontinuations occurred immediately after baseline.

**Conclusions:**

Extended depth of focus and MiSight® CL demonstrated similar efficacy in slowing myopia. When switched from a myopia control CL to SVCL, myopia progression was similar to that observed with age‐matched wearers in SVCL and not suggestive of rebound.

**Key points**

- With contralateral wear of myopia control contact lenses versus single‐vision contact lenses, myopia progressed slower in the eye wearing the myopia control lens in approximately 70% to 90% of participants.
- Myopia control mechanisms are localised to the eye receiving treatment, with no cross‐over effects to the contralateral eye wearing the single‐vision contact lens.
- Myopia progression after discontinuing thmyopia control contact lens was similar to the progression observed with a parallel, control group wearing single‐vision contact lenses, indicating no rebound of myopia.

## INTRODUCTION

The myopia epidemic has already arrived in many East Asian countries where the prevalence of myopia is over 70% in 17‐year olds; more alarming is the growing prevalence of high myopia in the region as it indicates a growing burden.^1^, ^2^ Furthermore, the global prevalence of myopia and high myopia is estimated to rise and portends a future increase in vision impairment and complications related to myopia.^3^, ^4^ The rising impact of myopia has ignited an interest in interventions to manage this burden, and in recent years multiple approaches involving optical and pharmaceutical strategies have been developed to slow myopia.^5^ These strategies are gaining momentum for use in mainstream clinical practice.^6^

Evidence for the effectiveness of myopia control strategies comes mostly from randomised clinical trials where myopic participants are allocated to one or more test or control groups and monitored for a defined period. The outcome/treatment effect is the difference in myopia progression between the means of the two distributions (test vs. control groups). The results of these trials have been widely reported and were also subject to meta‐analyses.^5^, ^7^, ^8^, ^9^ Although many trials report slower progression of myopia in the treated group compared with the control/untreated group, not every individual who receives the treatment will exhibit the mean treatment effect; whilst some individuals may have a treatment effect greater than the mean, others may have less or no effect. Furthermore, although randomised, there may be confounders that need to be managed when analysing the mean treatment effect. Therefore, it is difficult to quantify the benefit that a particular individual would receive from a given myopia control strategy.

Since myopia interventions are commonly bilateral, a contralateral study design provides an opportunity to determine whether a strategy has an effect for a given individual; one eye is used for treatment and the contralateral eye serves as a control. It is understood that such trials suffer from limitations such as induced anisometropia from refractive error manipulations, the possibility of consensual effects and dropouts due to inter‐eye differences in performance. However, when undertaken over a short period, they may be useful to screen potential treatments prior to embarking on long‐term studies, as they indicate the percentage of individuals likely to receive benefit, and are likely to require a smaller sample size than grouped long‐term, bilateral trials. Chua et al. in the 2006 ATOM 1 study used the contralateral eye as a control and monitored children for 1 year. Additionally, in a more recent study,^10^ Swarbrick et al.^11^ found that overnight orthokeratology inhibited eye growth when compared to conventional rigid gas permeable (RGP) contact lenses (CL).

Therefore, we conducted a randomised, prospective, clinical trial to determine the efficacy of two myopia control lenses: a novel extended depth of focus (EDOF) CL and a dual focus CL (MiSight®) in one eye compared with a single‐vision (SV) CL in the contralateral eye. To determine whether there were any consensual effects in the contralateral SV CL‐wearing eye, as well as to observe any carry‐over effects from switching between the lenses at the 6‐month time point, an additional group of children were randomised to wear bilateral single‐vision lenses for the entire 12‐month period.

## METHODS

In a prospective, masked, randomised clinical trial conducted at AIER Hospital group, Guangzhou, China, children with myopia aged 7–13 years, spherical equivalent refractive error ranging from −0.75 D to −3.50 D, astigmatism <0.75 D and anisometropia of ≤0.75 D were enrolled. All children had normal ocular findings with no strabismus, amblyopia, ocular pathology or any other findings that would prevent the participant from wearing contact lenses; additionally, they needed to have vision correctable to at least logMAR 0.20 (6/9.5) or better in each eye. Children with known allergies to cycloplegic or anaesthetic eye drops or a previous history of myopia control treatments were excluded. Parents/carers of children provided informed consent and indicated their willingness to attend the study visits as scheduled. The trial was approved by the Institutional Ethics Committee of AIER hospital network, adhered to the Declaration of Helsinki for experimentation on human subjects, and was conducted in accordance with the ICH‐GCP guidelines (ICH135/95). The trial was registered with the Chinese clinical trial registry (Chi‐CTR‐IOR‐17011993).

Enrolled children were randomised to three groups: Group I wore SV CL (control) in both eyes for 12 months; Groups II to III were randomised to either of the two test lenses (EDOF myopia control or MiSight®) in one eye and a SV CL in the contralateral eye. In Groups II and III, myopia control CL and SV CL were randomly allocated to the right and left eyes. The second stage (second 6 months) of the trial was a cross‐over to the first stage (first 6 months), participants in Groups II and III swapped the test and control CL between the eyes, that is, the eye that wore a test CL in the first 6 months swapped to the control SV CL and vice versa in the contralateral eye, and continued lens wear for a further 6 months.

### Lens design, study procedures and lens wear

All CL used in the study were hydrogel materials and worn on a daily wear, daily disposable schedule with lens wear for all waking periods and all days of the week. The control SV CL was a 1‐day Acuvue® Moist® (etafilcon A, Johnson & Johnson Vision Care Inc, jnj.com). The EDOF CL was a hydrogel Aquamax (etafilcon A, pegavision.com) that incorporated and manipulated selective higher‐order aberrations to achieve a through‐focus global retinal image quality that was optimised to degrade for points posterior to retina. The refractive power profile of the lens has been reported previously.^12^, ^13^ The MiSight® CL (Omafilcon A) has a central distance correction zone, with concentric peripheral zones alternating with the distance power and having a relative addition power of +2.00 D (coopervision.com).^14^

A baseline examination included history, visual acuity (high and low contrast), cycloplegic autorefraction, assessment of accommodative lag, heterophoria status and axial length measurements. Cycloplegia was achieved using 1% cyclopentolate hydrochloride (Alcon, www.alcon.com) preceded by a short‐acting anaesthetic proxymetacaine hydrochloride (alcon.com). Approximately 30 min following the instillation of the cycloplegic agent, the pupillary response to light was assessed prior to refractive error and axial length (AL) measurements. These parameters were assessed using the Shin Nippon K 5001 (shin‐nippon.jp) and Lenstar 900 (haag‐streit.com) instruments, respectively. Five and three measurements were recorded for autorefraction and axial length, respectively, and subsequently averaged. The CL power was chosen based on the cycloplegic spherical equivalent finding, followed by a spherical over‐refraction. The CL fit was assessed and participants trained in lens insertion and removal. Thereafter, participants were examined at 1, 3, 6, 9 and 12 months. Axial length was measured at baseline and every 3 months thereafter, whereas cycloplegic refractive error was measured at baseline and every 6 months thereafter. At each visit, participants rated their subjective visual performance for various aspects of lens wear (comfort, vision at far, intermediate and near, playing sport, lack of ghosting and haloes) on a scale from 1 to 10, where 10 = excellent and 1 = poor. At baseline, parents completed a questionnaire that gathered information regarding the age at which myopia was diagnosed (if known), parental myopia and the number of hours spent each day reading, using digital devices and outdoor activities during typical school terms and holidays. Accommodative lag and heterophorias (distance and near) were measured at 6‐monthly intervals.

Participants were required to apply the CL on waking, wear them for all waking hours and dispose of them at the end of the day prior to sleep. Participants were advised not to sleep in the lenses overnight. Short naps in the CL during the daytime were allowed. All children were required to have an up‐to‐date pair of spectacles for any occasions where they were unable to wear CLs. Whilst participants could dispose of their own CLs, they were required to collect and bring in the foils of the lens blister packs for each lens worn to check for compliance. Since CL were used on a daily disposable schedule, no disinfecting solutions were dispensed. Participants were advised to use unit dose saline (0.9% sodium chloride, pfizer.com) as necessary for lens rinsing, as an in‐eye lubricant or for storing lenses removed due to an adverse event.

### Data analysis

Prior CL studies demonstrated a 6‐monthly AL progression of approximately 0.13 to 0.19 mm.^12^, ^15^ Using a progression of 0.16 (SD = 0.11) mm, a sample of 30 participants per group was required to detect a 40% reduction with the test lens compared with the contralateral control lens at a 5% level of significance and 80% power for a 2‐tailed distribution. The estimated sample size was adjusted for a 20% dropout rate. The required sample also had 80% power to detect a paired difference in progression of spherical equivalent refractive error of 0.16 ± 0.25 D, which represents a 40% reduction with the test lens compared with historical control lens progression.^12^

Spherical equivalent refractive error and AL were recorded on an interval scale. The spherical equivalent was computed as sphere + half cylinder power. Progression was defined as the changes in refractive error and AL from the dispensing visit, computed at each stage for each participant eye and summarised as means ± standard deviations. Six‐monthly progression across both stages was analysed using a linear mixed model with lens type, stage of the trial, eye and interaction of eye and stage included as factors. Stages and eyes were factored as repeated effects with compound symmetry covariance structure, and intra‐participant correlation was accounted for by using participant random intercepts. If interaction was significant, then the effect of lens type across each stage was analysed. Model‐based estimated means and their 95% CI were reported, and *post*‐*hoc* multiple comparisons were adjusted using Bonferroni correction. Visual acuity and subjective ratings were compared between study groups at each visit using paired *t*‐tests, and *p*‐values were adjusted using Bonferroni correction.

With groups II and III, test eyes that progressed less (any contralateral difference) than the control eyes were defined as ‘responders’ and summarised as a percentage of participants at the 6‐ and 12‐month visits. The percentage of responders was compared between test lens types using a generalised linear model. With respect to non‐responders (same or greater progression in the test eyes compared with the control eyes at one or both stages), a univariate analysis was conducted to determine the association, if any, with responders in one or more stages for demographic, ocular parameters and environmental risk factors. The level of significance was set at 5%. SPSS software (v27, ibm.com) was used for statistical analysis.

## RESULTS

A total of 95 children were successfully enrolled and randomised to Group I (bilateral single vision, *n* = 30), Group II (EDOF vs. single vision, *n* = 31) and Group III (MiSight® vs. single vision, *n* = 34). Results are shown throughout with the standard deviation (SD) in parentheses.

Table 1 details the baseline data of the enrolled participants, but in brief, the mean age of children was 10.8 (1.5) years, mean spherical equivalent refractive error was −1.99 (0.68) D and the mean axial length was 24.6 (0.8) mm. There were no significant differences in these parameters between the groups.

**TABLE 1:** Baseline data of all participants who enrolled and for participants that completed the 6‐month visit

Variable	*n*	SV CL (Group I)	*n*	EDOF (Group II)	*n*	MiSight® (Group III)	*p*‐Value
All enrolled participants							
Age in years Mean (SD) Range	30	10.9 (1.5) 7.9 to 13.4	31	10.8 (1.5) 7.7 to 13.6	34	10.8 (1.6) 7.0 to 13.4	0.97
Female: Male (%)	30	43:57	31	55:45	34	53:47	0.63
Parental myopia % None: One: Two	30	33.3:43.3:23.3	30	36.7:40.0:23.3	32	28.1:37.5:34.4	0.84
Cycloplegic SE (D) Mean (SD), Range	60	−2.08 (0.64) −1.10 to −3.83	60	−2.01 (0.68) −0.90 to −3.30	66	−1.91 (0.72) −0.71 to −3.61	0.34
Axial length (mm) Mean (SD); Range	60	24.5 (0.6) 23.3 to 25.5	62	24.6 (1.0) 23.1 to 27.7	66	24.5 (0.8) 23.2 to 27.0	0.96
Participants completed 6‐month visit							
Age in years Mean (SD) Range	21	11.0 (1.5) 7.9 to 13.4	19	10.7 (1.4) 8.2 to 13.6	21	11.2 (1.7) 8.1 to 13.4	0.58
Female: Male (%)	21	43:57	19	63:37	21	67:33	0.24
Parental myopia% None: One: Two	21	33:43:24	19	37:47:16	21	29:33:38	0.61
Cycloplegic SE (D) Mean (SD), Range	42	−2.10 (0.66) −1.10 to −3.83	38	−1.89 (0.62) −0.94 to −3.16	42	−1.97 (0.73) −0.71 to −3.61	0.34
Axial length (mm) Mean (SD); Range	42	24.60 (0.59) 23.4 to 25.5	38	24.31 (0.74) 23.1 to 26.1	42	24.47 (0.79) 23.2 to 26.2	0.21

Abbreviations: EDOF, extended depth of focus lens; SD, standard deviation; SE, spherical equivalent refractive error.

Of the 95 participants who were dispensed with lenses, 42 discontinued before completing the study. Although the discontinued participants were younger, there were no differences between completed versus discontinued participants for baseline cycloplegic refractive error or AL (Table 2). Most discontinuations occurred immediately after baseline and prior to the one‐month visit (26/42 or 62%), whilst the remainder occurred throughout the trial (16/42). Discontinuations were 33.3%, 48.4% and 50.0% for groups I to III, respectively, and did not differ significantly between the groups (*p* = 0.35; additionally, group II and III discontinuations were not higher than group I discontinuation [*p* = 0.19]). Since most discontinuations occurred immediately after baseline, the baseline data for participants who presented for and completed the 6‐month visit were compared (Table 1) and show no differences in characteristics across the three groups. The reasons for discontinuations were as follows: not comfortable with lens wear or not using lenses regularly (11/42), no longer interested in lens wear (9/42), issues with lens handling (9/42), lost to follow up (6/42), time issues (2/42), safety concerns (3/42), other symptoms (1/42) and dissatisfied with the quality of vision through the contact lens (1/42).

**TABLE 2:** Age and ocular characteristics of participants who completed all study visits versus those who discontinued before the end of the trial

	Completed	Discontinued	*p*‐Value
Age in Years; Mean (SD)	11.2 (1.5)	10.4 (1.4)	0.009
Female: Male (%)	55:45	45:55	0.41
Cycloplegic SE (D) Mean (SD)	−2.00 (0.70)	−1.99 (0.66)	0.59
Axial length (mm) Mean (SD)	24.50 (0.71)	24.64 (0.96)	0.17

Abbreviations: SD, standard deviation; SE, spherical equivalent refractive error.

At baseline, paired differences between the right and left eyes did not differ significantly for spherical equivalent refractive error (−0.05 (0.38) D, −0.09 (0.42) D and −0.02 (0.41) D for groups I to III, respectively) or axial length (0.0 (0.2) mm, 0.0 (0.1) mm and 0.0 (0.2) mm for groups I to III, respectively).

### Stage 1: Baseline to 6 months: Change in spherical equivalent refractive error and axial length

**FIGURE 1:**
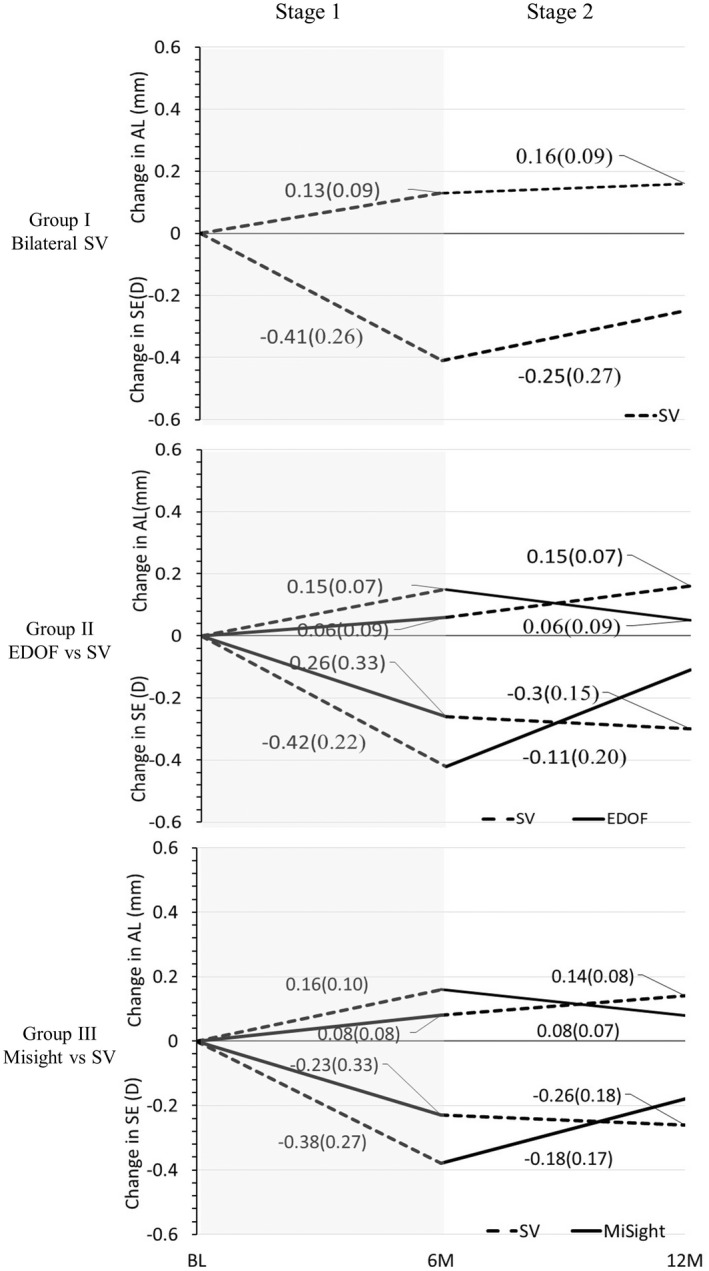
Change in spherical equivalent and axial length (mean (SD)) for groups I to III for Stage 1: Baseline (BL) to 6 months (6 M) and Stage 2: 6 months to 12 months; the effect of cross‐over at 6 months is demonstrated. EDOF, extended depth of focus; SV, single vision.

In group I, the change in spherical equivalent/axial length during Stage 1 was −0.41 (0.30) D/0.12 (0.10) mm in the right eyes and −0.41 (0.28) D/0.13 (0.09) mm in the left eyes (both eyes combined −0.41 [0.28] D/0.13 [0.09] mm) (Figure 1a). The paired differences were not significant for either the change in spherical equivalent (0.01 [0.17] D, *p* = 0.85) or axial length (0.01 [0.05] mm, *p* = 0.48).

In group II, EDOF CL‐wearing eyes progressed slower compared with the contralateral eyes wearing SV CL (Figure 1b); this was consistent for both the change in spherical equivalent and axial length. The paired difference was 0.16 (0.24) D, and −0.10 (0.06) mm, with 39% and 63% less change with EDOF for spherical equivalent and axial length, respectively.

Similarly, in group III, MiSight® CL‐wearing eyes progressed slower than the contralateral SV CL‐wearing eyes (Figure 1c). The paired difference was 0.16 (0.32) D, (41% less change with MiSight® lens) and −0.08 (0.08) mm, (48% less change with MiSight® lens) for spherical equivalent and axial length, respectively.

The control SV CL‐wearing eyes from Groups II and III progressed similarly to those wearing bilateral SV CL in Group I; the mean change in spherical equivalent/axial length over the 6‐month period was similar between the groups.

### Stage 2: 6 months to 12 months: Change in spherical equivalent and axial length

In group I, the change in SE/AL was slower during Stage 2 compared with Stage 1 at −0.27 (0.27) D/0.16 (0.09) mm and −0.22 (0.27) D/0.16 (0.09) mm in the right and left eyes, respectively (both eyes combined −0.25 (0.27) D/0.16 (0.09) mm, Figure 1a). The paired differences between the eyes were not significant for either the change in SE (0.05 [0.19] D, *p* = 0.27) or AL (−0.01 [0.05] mm, *p* = 0.64).

In groups II and III, eyes that crossed over from wearing control SV CL wear in Stage 1 to either EDOF or MiSight® lenses during Stage 2 slowed in progression. In comparison, when switched from myopia control CL to SV CL, the rate of progression increased, but was similar to and not greater than that observed for SV CL‐wearing eyes from group I.

In group II, the change in SE and AL was slower with the EDOF CL compared with the contralateral control SV CL (Figure 1b). The paired difference for SE and AL was 0.20 (0.20) D, (64% less change with EDOF CL) and −0.11 (0.08) mm (66% less change with EDOF CL), respectively.

Similarly, in group III, the change in SE and AL was slower with the MiSight® CL compared with the contralateral SV CL (Figure 1c). The paired difference for SE and AL was 0.08 (0.21) D (30% less change with MiSight® CL) and −0.06 (0.09) mm (42% less change with MiSight® CL), respectively.

The model‐based estimated change in SE and AL for each lens type is provided in Table 3. The model included lens type, stage of the trial and interaction of lens type*stage. Overall, the 6‐month progression with both MiSight® and EDOF was significantly lower than for SV control lenses for both change in AL (linear mixed model, *p* < 0.001) and SE (*p* = 0.009, 0.001 for MiSight® and EDOF, respectively). The change in AL was not significantly different between Stages 1 and 2 (*p* = 0.43), whereas the change in SE was significantly lower during Stage 2 (*p* = 0.002). The interaction between lens types and both changes in AL (*p* = 0.79) and SE (*p* = 0.51) were not significant.

**TABLE 3:** Model‐based estimated mean change in spherical equivalent and axial length across the stages

Lens type	Stage 1		Stage 2		Stages 1 and 2 combined	
	Mean	95% CI	Mean	95% CI	Mean	95% CI
Estimated change in axial length (mm)						
SV control	0.14	0.12 to 0.16	0.16	0.14 to 0.18	0.15	0.13 to 0.17
EDOF	0.05	0.02 to 0.09	0.05	0.02 to 0.09	0.05	0.02 to 0.08
MiSight®	0.08	0.05 to 0.12	0.09	0.05 to 0.13	0.08	0.06 to 0.11
Estimated change in spherical equivalent (D)						
SV control	−0.41	−0.46 to −0.35	−0.27	−0.33 to −0.21	−0.34	−0.39 to −0.29
EDOF	−0.24	−0.35 to −0.14	−0.10	−0.22 to +0.01	−0.17	−0.26 to −0.09
MiSight®	−0.24	−0.34 to −0.14	−0.19	−0.30 to −0.08	−0.21	−0.30 to −0.13

*Note:* Model included lens type, Stage of the trial and interaction of Lens type*Stage.

Abbreviations: CI, confidence interval; EDOF, extended depth of focus lens; SV, single vision.

### Stages I and II combined: Paired data comparison

**FIGURE 2:**
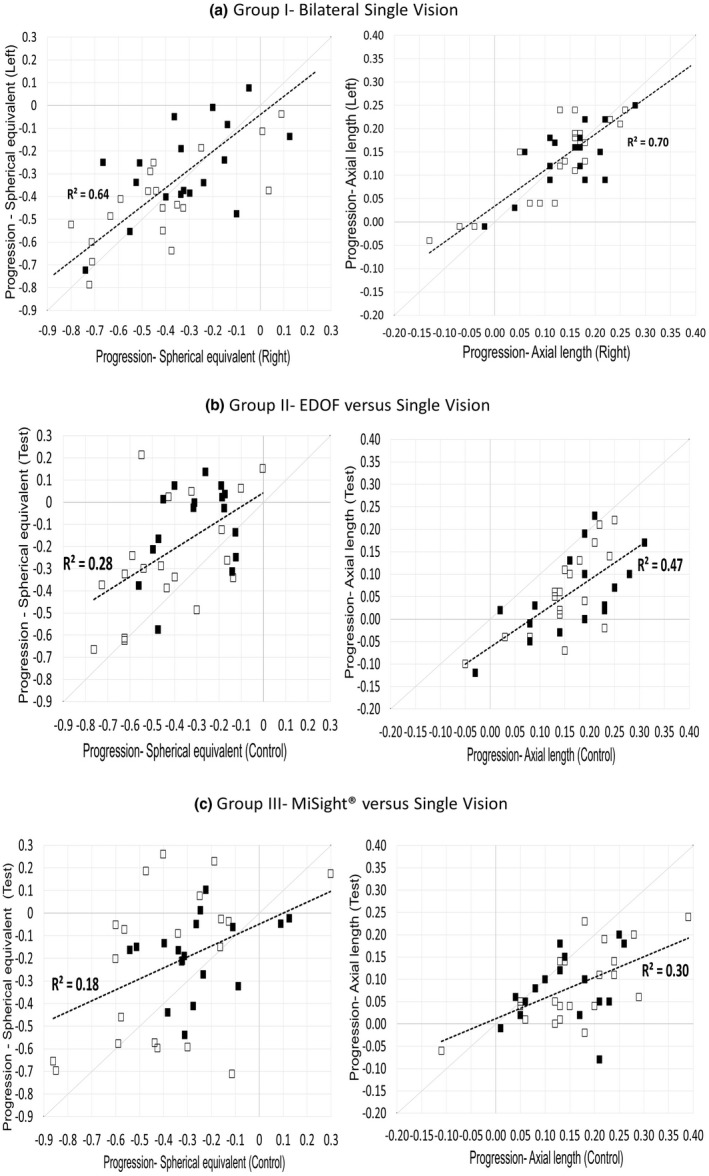
Paired differences between eyes of an individual for both stages of Group I (a), Group II (b), Group III (c) for change in spherical equivalent and axial length (Open filled markers—first stage; dark filled markers—second stage; continuous grey line through [0, 0]—line of no difference; dashed dark line—best fitting linear fit with *R* ^2^ value). Abbreviation: EDOF, extended depth of focus lens.

**FIGURE 3:**
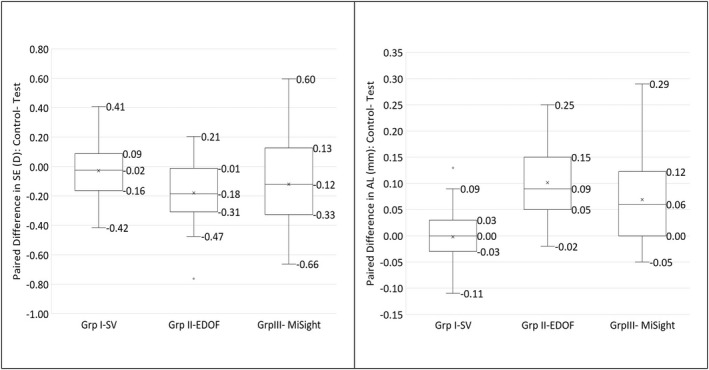
Box plots demonstrating paired differences between eyes across Groups I to III for change in spherical equivalent (SE) and change in axial length (AL). EDOF, extended depth of focus lens; SV, single‐vision contact lens. The provided values indicate the median (mid value) and the range (top and bottom values). The asterisk denotes the mean and the open circle (_°_) outside the range denotes outliers.

Figures 2 and 3 further illustrate the correlation and paired differences between the eyes of an individual across the groups. In group I, the correlation between the right and left eyes was high (Figure 2a) with negligible differences between paired eyes (Figure 3). In comparison, the correlation between test and control eyes was lower (Figure 2b,c) and paired differences greater for groups II and III.

In group II, 77% to 94% of participants responded with reduced progression (change in SE and AL, respectively) with the EDOF CL compared with the control SV CL. Similarly, 68% to 76% of group III participants showed reduced progression (change in SE and AL, respectively) with the MiSight® CL compared with the SV CL. The proportion of participants responding to either the EDOF or MiSight® CL was not significantly different for the change in SE (*p* = 0.41) but was significant for change in AL (*p* = 0.04). However, across both stages, the adjusted 6‐month myopia control efficacy of the EDOF was not significantly different from MiSight® (−0.16 D, CI: 0.05–0.26 D vs. −0.10 D, CI: 0.00–0.20 D, *p* = 0.45) for SE and (−0.08 mm, CI: −0.05 to −0.13 mm vs. −0.07 mm, CI: −0.04 to −0.10 mm, *p* = 0.56) for AL.

Table 4 presents an analysis of risk factors between non‐responders (test lens‐wearing eyes with similar or greater progression than control eyes) and responders. No significant differences were observed between the groups.

**TABLE 4:** Univariate analysis of risk factors between non‐responders and responders

	Non‐responders		Responders (one stage)		Responders (both stages)		*p*‐Value
	*n*	Value	*n*	Value	*n*	Value	
Age at consent (years)	6	10.8 (1.1)	15	10.5 (1.9)	19	11.4 (1.3)	0.29
Gender (M/F %)	6	33.3/66.7	15	80/20	19	63.2/36.8	0.13
Parental myopia % (none/one/both)	6	16.7/66.7/16.7	15	26.7/33.3/40.0	19	42.1/36.8/21.1	0.43
Age myopia detected (years)	6	9.0 (1.1)	14	9.0 (1.6)	19	9.2 (1.5)	0.18
Axial length (mm), eyes	12^a^	24.80 (0.68)	30^a^	24.22 (0.75)	38^a^	24.41 (0.77)	0.08
Spherical equivalent (D), eyes	12^a^	−2.08 (0.80)	30^a^	−2.00 (0.62)	38^a^	−1.82 (0.68)	0.41
Reading and writing at school (h)^b^	6	3.0 (1.1)	15	2.0 (1.3)	19	3.2 (1.7)	0.09
Reading and writing at home (h)^b^	6	1.8 (1.0)	15	1.9 (1.0)	19	1.7 (1.2)	0.83
Outdoors at school (h)^b^	6	1.3 (0.5)	15	0.9 (0.3)	19	1.1 (0.4)	0.23
Outdoors at home (h)^b^	6	0.3 (0.4)	15	0.6 (0.8)	19	0.7 (1.0)	0.58
Primary gaze movement (mm), all visits	28	0.24 (0.17)	65	0.27 (0.29)	87	0.29 (0.21)	0.54
Lens tightness (subjective push up test, 1–100%, 1 = extremely loose, 100 = tight), all visits	28	49.5 (3.6)	65	49.0 (3.0)	87	49.2 (3.4)	0.84

^a^ Eyes, all other refer to participants.

^b^ Collected at the baseline visit.

### Visual performance in groups II and III

High‐contrast visual acuity with the EDOF CL in group II and MiSight® lens in Group III did not differ significantly from the visual acuity achieved with the control SV lenses. (Table 5). With regard to subjective vision clarity overall, both groups rated their vision to be near excellent with slightly lower rating for the EDOF and MiSight® lenses compared with the controls at 3 months.

**TABLE 5:** Visual performance EDOF: Extended depth of focus contact lens, Control: single‐vision contact lens

Parameter	Visit (months)	Group II			Group III		
		EDOF (Mean (SD))	Control (Mean (SD))	*p*‐Value	MiSight® (Mean (SD))	Control (Mean (SD))	*p*‐Value
Presenting high‐contrast VA (logMAR)	3	0.26 (0.24)	0.22 (0.26)	0.10	0.12 (0.23)	0.15 (0.27)	>0.99
	6	0.34 (0.27)	0.30 (0.33)	0.61	0.17 (0.29)	0.20 (0.29)	0.44
	9	0.16 (0.15)	0.05 (0.15)	0.004	0.13 (0.22)	0.13 (0.23)	>0.99
	12	0.21 (0.24)	0.14 (0.27)	0.11	0.26 (0.33)	0.26 (0.35)	>0.99
Vision clarity (1–10)	3	9.2 (1.2)	9.4 (1.1)	0.08	9.5 (1.0)	9.9 (0.4)	0.17
	6	9.2 (1.4)	9.1 (1.8)	>0.99	9.5 (0.9)	9.5 (0.8)	>0.99
	9	9.4 (1.2)	9.5 (0.9)	>0.99	8.6 (2.1)	9.3 (1.6)	0.28
	12	9.3 (0.8)	9.5 (0.6)	>0.99	9.5 (0.8)	9.8 (0.4)	0.68

## DISCUSSION

The contralateral study design demonstrated that both EDOF and MiSight® lenses produced a significant slowing of myopia. Beyond the demonstration of myopia control efficacy, there were other important findings. Firstly, during each stage, myopia progression in the SV lens‐wearing eyes was similar across all groups. This suggests that there was no consensual or cross‐over effect for growth in the contralateral eye. Previous studies in humans have demonstrated that myopic defocus in one eye slows myopia; however, whether this has implications for the growth of the contralateral eye remained uncertain.^11^, ^16^ Consensual effects are commonly observed for a range of ocular states/conditions. Instillation of a mydriatic drug into one eye results in a dose‐dependent constriction of the fellow eye due to increased light reaching the retina and sending afferent signals to the third nerve nuclei, thereby evoking a parasympathetic response.^17^ Similarly, the accommodative response is similar between seeing and non‐seeing eyes or in eyes of isometropic individuals subject to accommodative demands in only one eye.^18^, ^19^ In contrast, the factors that influence eye growth are considered to be largely independent between the two eyes—there is a vast body of evidence from both animal and human studies demonstrating that monocular manipulations predictably alter refractive error and AL in the eye that is subject to manipulation.^20^ Therefore, it is suggested that the mechanisms that regulate refractive development are local and operate in a regionally selective manner and independently of accommodation.^21^ Whilst the underlying mechanisms regulating growth remain unknown, these data support and indicate that the changes are entirely localised to the eye receiving treatment.

Another important observation relates to myopia progression in the eyes that switched from myopia control lenses at the end of Stage 1 to SV lenses in Stage 2. Here, during stage 2, myopia progression was similar to that observed in Group I SV lens wearers. In the ATOM 1 study, following 2 years of atropine use in one eye, progression was significantly higher or ‘rebounded’, especially in the first 6 months after discontinuation, in comparison with the contralateral eye.^22^, ^23^ Using a contralateral design similar to the current study, progression in a rigid gas permeable CL following 6 months of orthokeratology treatment appeared to be faster and was considered to represent a ‘rebound effect’.^11^ In the current trial, we compared the progression that occurred after discontinuation of the myopia control CL over the second 6 months with the rate of progression observed in the Group I single‐vision lens‐wearing eyes over the same period. The data indicate that although discontinuation from myopia control lenses leads to an increase in progression, the rates of progression were similar to those observed with single‐vision lenses in Group I and, therefore, not supportive of a rebound effect.

The paired data analysis found that the mean difference in progression for group I eyes across both stages was 0.02 D and 0 mm for SE and AL, respectively, or in other words, the progression between the right and left eyes was highly correlated. In group II and group III eyes, the paired difference in progression between the eyes was significantly different with a lesser myopic shift in the test lens‐wearing eyes. In group II, across both stages, 77% to 94% of participants were EDOF responders based on the change in SE and AL, respectively. Similarly, in group III, 68% to 76% of participants were MiSight® responders based on the change in SE and AL, respectively. Although there were more responders to EDOF CL based on AL, overall, most eyes responded to myopia control lenses with similar effects between the lens types. We tried to determine whether there were risk factors such as age of myopia detection, lifestyle or ocular factors that were associated with non‐responsiveness to myopia control lenses but found no significance with any factors. However, caution needs to be exercised with this analysis due to the low power of the sample.

Unlike the current trial where both myopia control lenses demonstrated similar performance, in long‐term dispensing trials, the EDOF lens was observed to slow the progression of myopia by up to 32% for SE and 25% for AL at the end of 2 years.^12^ In contrast, the MiSight® lens slowed myopia by up to 59% for SE and 52% for AL.^14^ The reasons for this difference are not clear, but multiple factors such as study methodology, compliance to lens wear and other unknown factors may be responsible.

It is well known that age influences myopia progression.^24^, ^25^ For the group with bilateral SV lenses, myopia progressed by −0.41 D/0.13 mm during the first 6 months and − 0.25 D/0.16 mm during the second 6 months. It has previously been reported that there may be tapering efficacy over time, and this may be related to slowing axial elongation that makes it more difficult to detect differences between the groups.^26^ In the current trial, the efficacy of the myopia control lenses remained high and equally effective in the second 6‐month period as during the first 6 months. Whilst this indicates that the CL are effective even in eyes with slower progression, it should be noted that the sample size was limited and in situations where efficacy is observed to be tapering, usually the same treatment is continued, whereas in the present study, the lenses were switched after 6 months of lens wear. Therefore, the results are not directly comparable.

The present study suffers from some significant limitations. There were many discontinuations from the trial. The contralateral study design induces an imbalance in binocular vision in the initial phase and anisometropia towards the end of the wearing stage. Although our vision measurements and subjective performance assessments did not uncover significant issues, we acknowledge the limitations of measurements such as high‐contrast visual acuity, and it is possible that the greater number of dropouts from the test groups, especially those occurring early in the study, may be reflective of vision performance issues. Also, we were unable to explore whether, for example, the eyes with multifocal lenses accommodated appropriately to near tasks. In a previous study, under‐correction in one eye slowed myopia. The eye corrected for distance was found to have accommodated when viewing near targets, and therefore, the under‐corrected eye remained in myopic defocus at all times.^16^ Such assessments are difficult with multifocal lens wear.

In summary, both EDOF and MiSight® myopia control lenses were equally effective in slowing myopia. Most eyes responded to the anti‐myopigenic stimulus imparted by these lenses, and furthermore, upon discontinuation, the progression was similar to that with conventional single‐vision CL.

## AUTHOR CONTRIBUTIONS

**Rebecca Weng:** Conceptualization (supporting); investigation (supporting); project administration (lead); writing – original draft (equal). **Weizhong Lan:** Investigation (equal); project administration (equal); resources (equal); supervision (equal); writing – review and editing (equal). **Ravi Bakaraju:** Conceptualization (equal); writing – review and editing (equal). **Fabian Conrad:** Conceptualization (equal); writing – review and editing (equal). **Thomas Naduvilath:** Conceptualization (equal); data curation (equal); formal analysis (equal); methodology (equal); visualization (equal); writing – review and editing (equal). **Zhikuan Yang:** Resources (equal); supervision (supporting); writing – review and editing (supporting). **Padmaja Sankaridurg:** Conceptualization (equal); data curation (equal); formal analysis (equal); supervision (equal); visualization (equal); writing – original draft (equal); writing – review and editing (equal).

## Disclosures

RW, WL, RB‐ P, FC‐P, TN‐P, ZY declare that they have no conflict of interest. PS has commercial interests in myopia control.
